# Vaccinia virus expressing bone morphogenetic protein-4 in novel glioblastoma orthotopic models facilitates enhanced tumor regression and long-term survival

**DOI:** 10.1186/1479-5876-11-155

**Published:** 2013-06-24

**Authors:** Rohit Duggal, Ulrike Geissinger, Qian Zhang, Jason Aguilar, Nanhai G Chen, Elena Binda, Angelo L Vescovi, Aladar A Szalay

**Affiliations:** 1 Genelux Corporation, San Diego Science Center, 3030 Bunker Hill Street, Suite 310, San Diego, CA 92109, USA; 2 Department of Biochemistry, Biocenter, University of Würzburg, Am Hubland, 97074, Würzburg, Germany; 3 Department of Radiation Oncology, Moores Cancer Center, University of California, 3855 Health Sciences Drive, La Jolla, San Diego, CA 92093, USA; 4 Department of Biotechnology and Biosciences, University of Milano-Bicocca, Piazza della Scienza 2, I-20126, Milano, Italy

**Keywords:** Vaccinia virus (VACV), Glioblastoma multiforme (GBM), Bone morphogenetic protein (BMP), Cancer stem cells (CSCs) and differentiation

## Abstract

**Background:**

Glioblastoma multiforme (GBM) is one of the most aggressive forms of cancer with a high rate of recurrence. We propose a novel oncolytic vaccinia virus (VACV)-based therapy using expression of the bone morphogenetic protein (BMP)-4 for treating GBM and preventing recurrence.

**Methods:**

We have utilized clinically relevant, orthotopic xenograft models of GBM based on tumor-biopsy derived, primary cancer stem cell (CSC) lines. One of the cell lines, after being transduced with a cDNA encoding firefly luciferase, could be used for real time tumor imaging. A VACV that expresses BMP-4 was constructed and utilized for infecting several primary glioma cultures besides conventional serum-grown glioma cell lines. This virus was also delivered intracranially upon implantation of the GBM CSCs in mice to determine effects on tumor growth.

**Results:**

We found that the VACV that overexpresses BMP-4 demonstrated heightened replication and cytotoxic activity in GBM CSC cultures with a broad spectrum of activity across several different patient-biopsy cultures. Intracranial inoculation of mice with this virus resulted in a tumor size equal to or below that at the time of injection. This resulted in survival of 100% of the treated mice up to 84 days post inoculation, significantly superior to that of a VACV lacking BMP-4 expression. When mice with a higher tumor burden were injected with the VACV lacking BMP-4, 80% of the mice showed tumor recurrence. In contrast, no recurrence was seen when mice were injected with the VACV expressing BMP-4, possibly due to induction of differentiation in the CSC population and subsequently serving as a better host for VACV infection and oncolysis. This lack of recurrence resulted in superior survival in the BMP-4 VACV treated group.

**Conclusions:**

Based on these findings we propose a novel VACV therapy for treating GBM, which would allow tumor specific production of drugs in the future in combination with BMPs which would simultaneously control tumor maintenance and facilitate CSC differentiation, respectively, thereby causing sustained tumor regression without recurrence.

## Background

The emergence of a cancer stem cell (CSC) concept has if not revolutionized but certainly altered views about the origin(s) of cancer and what the new anti cancer modalities should target. The main properties of CSCs as identified by a distinguished group of CSC scientists after the AACR workshop in 2006 [1] are the ability to initiate and maintain a tumor including the CSC compartment (self renewal) and generation of differentiated progeny that make up the bulk of the tumor. This makes the CSC at the apex of neoplastic transformation where its unique stem cell properties of self renewal and multipotency enables it to initiate, fuel and sustain tumor growth. The original study by John Dick and colleagues that used immunodeficient mice to xenograft tumorous cells was a seminal study [2]. These researchers found that most subtypes of acute myeloid leukemia could be implanted in these mice, but found heterogeneity within these tumors. Only one in a million tumor cells could initiate tumors, thereby this capability lying in only a subset of tumorous cells. In case of solid tumors, the ground breaking work was carried out by Clarke and coworkers in 2003 [3]. They established the tumor initiating capability to reside in a subset of cells in breast tumors. This was followed by identification of CSCs in brain tumors [4,5]. Very interestingly it was demonstrated that the GBM CSCs are multipotent and could be maintained as spheroids *in vitro* almost indefinitely without significant change in properties [4]. CSCs have also been identified now in colon cancer, pancreatic cancer, liver cancer, ovarian cancer, melanoma and thyroid cancer [6-12].

Initial efforts for targeting CSCs involved targeting pathways that are involved in development that are thought to be active in undifferentiated and primitive cells, namely the Wnt-beta catenin, Notch and the Hedgehog pathways [13-15]. Limited success has been achieved targeting these pathways using small and large molecule inhibitors [16-18]. Another class of therapeutics entails the use of recombinant proteins that are being designed exclusively to target the undifferentiated cell population component in tumors ( [19,20]. Prominent among this class has been recombinant proteins belonging to the TGF-beta superfamily of proteins called bone morphogenetic proteins (BMPs). BMPs are involved in embryonic development, organ morphogenesis and adult tissue homeostasis [21,22]. There is direct and indirect evidence for a role of BMPs in regulating cancer. Mutations in the BMP receptor or Smad4, a key mediator of BMP signal transduction predisposes patients to colorectal cancer [23,24]. It is also documented that upregulation of inhibitors of BMPs, such as Coco and Gremlin result in activation of breast cancer metastases [25] and occurrence of lung adenocarcinoma [26], respectively. In the context of gliomas, BMP-4 expression was found to correlate well with lower grade gliomas and better prognosis in grade III and grade IV gliomas (GBM) [27]. BMPs have also been shown to inhibit breast CSCs and the tumorigenicity of an osteosarcoma cell line [28,29]. Practical application of BMPs and their ability to negatively regulate cancer has come from the work of Piccirillo, et al., where they have shown BMPs can cause rapid tumor regression in case of GBM [19] and made a case for use of BMPs in the treatment of the disease. More recently BMP-4 has been used as a differentiation agent in controlling colon cancer in mice [30] using models based on CSCs.

There are few literature reports of studies involving CSCs and infections by oncolytic poxviruses. Vaccinia virus, a member of the family poxviridae has been found to not infect all primary hematolymphoid cells [31,32]. Therefore, there could be a tropism issue associated with infection of primary cells by vaccinia virus that could be accentuated upon using attenuated mutants used for oncolytic therapy. However, some other poxviruses, such as myxoma virus has been shown to readily infect primary neuroblastoma CSCs. Therefore, it has been of interest to test oncolytic vaccinia viruses against bonafide CSC preparations to determine susceptibility to infection. We had hypothesized that expressing payloads such as BMPs from oncolytic vaccinia viruses would facilitate delivery of the proteins to expedite differentiation of previously validated CSCs that produce GBM in an authentic manner [4]. Indeed, here we report, that BMP-4 expressing vaccinia viruses produce the protein in primary GBM cultures and in the brains of GBM CSCs-transplanted mice, differentiate GBM CSCs and further increase replication capacity of the virus resulting in substantial tumor regression and survival benefit to mice implanted with the GBM CSCs.

## Methods

### Cell culture

The primary GBM CSC cultures were derived from tumor biopsies and labeled based on the day the biopsy was obtained, with the first two digits standing for the year, the next two for the month and the last two for the day. These cultures were propagated under serum-free conditions as described previously [4]. Briefly, these cultures were propagated in Neurocult NS-A medium (Stem Cell) in the presence of EGF and b-FGF (NSC medium) [4]. U87, U373 and U251 glioma lines were obtained from the ATCC. They were grown based on the recommendations of the supplier. In order to adapt the glioma cell lines to stem cell conditions, the cell lines were passaged under conditions as described above and a suffix “s” added after name of each cell line. All cell lines were authenticated by morphology and growth characteristics. To create a firefly luciferase (FLuc) expressing U87 cell line, U87 cells were transfected with a plasmid that expresses the FLuc cDNA (pGL4.51[luc2/CMV/Neo], Promega) using Lipofectamine (Life Technologies). The stable cell line was selected with 500 μg/mL G418 sulfate (Mediatech).

### Construction of recombinant VACV strains expressing BMP-4

A cDNA encoding the human BMP-4 was PCR amplified using Human Universal cDNA mix (Clontech) as the template with primers BMP-4-5 (5’-GTCGAC(*Sal I*) CACCATGATTCCTGGT AACCGAATGCTGATGG -3’) and BMP-4-3 (5’-TTAATTAA(*Pac I*) TCAGCGGCACCCACATCC -3’). The PCR product was gel-purified and cloned into the pCR-Blunt II-TOPO vector using Zero Blunt TOPO PCR Cloning Kit (Invitrogen). The sequence of BMP-4 cDNA was confirmed and was released with *Sal I* and *Pac I* digestion and subcloned into the vaccinia TK transfer vectors cut with the same restriction enzymes, placing the BMP-4 cDNA under the control of the early/late VACV promoter. The resulting constructs were used to make recombinant virus, GLV-1h285 using GLV-1h189 as the parental virus as previously described [33]. BMP-4 expression from GLV-1h285 was confirmed by western blot analyses where both the secreted and precursor forms were detected upon infecting GBM CSCs and CV-1 cells (data not shown).

### Cell growth inhibition and virus replication assays

Cell growth inhibition assays were carried out in 96 well black plates (Costar). Eight serial virus dilutions (1:3) were carried out to keep the concentration twice that of the final concentration. A 100 μL sample of each cell line (1.7 × 10^5^ cells/mL) was mixed with 100 μL of each virus dilution and 30 μL of this was plated in triplicate for each cell line. Virus adsorption was carried out at 37°C for an hour and then the volume was brought up to 150 μL with NSC medium. At day 9, plates were developed using the Cell titer glo (CTG) kit (Promega) and read with a SpectraMax M5 plate reader (Molecular Dynamics). The effective concentration (EC_50_) values were calculated as the virus multiplicity of infection (MOI) at which 50% growth inhibition was achieved.

Replication assays were carried out as the growth inhibition assays except that the *Renilla* luciferase (RLuc) glo kit (Promega) was employed. To determine that BMP-4 increased replication of GLV-1h285, GBM CSC line 010627 was infected with GLV-1h189 in the presence of 100 ng/mL of purified BMP-4 (R&D Systems) and replication was measured by RLuc expression at day 9 post infection (dpi). For determining viral titers, GBM CSC line, 010627 and U87s were infected at an MOI of 0.25 with both GLV-1h189 and GLV-1h285. Cultures were collected 9 dpi and subjected to 3 freeze-thaw cycles. Virus plaque assays were carried out as previously described [34].

### Immunofluorescence staining

Cells of GBM CSC line, 010627 line were seeded on laminin (Roche) coated 24 well plates and treated with 100 ng/mL BMP-4 (R&D systems) or were infected with viruses at an MOI of 1. After 4 days samples were fixed in 4% methanol-free paraformaldehyde in PBS and permeabilized with 0.25% Triton X-100. To block nonspecific binding of the antibodies cells were incubated with 1% BSA in PBS Triton X-100 (PBST) for 30 minutes. Cells were incubated with primary antibody against glial fibrillary acidic protein (GFAP, Dako Cytomation) diluted 1:500 in 1% BSA in PBST in a humidified chamber for 1 hour at room temperature. The secondary antibody (Alexa Fluor® 350 goat anti-rabbit IgG, Invitrogen) was diluted 1:500 in 1% BSA and incubated for 1 hour at room temperature in the dark. The plates were observed under a fluorescence microscope (Carl Zeiss) and photographed.

### Intracranial tumor cell implantation and inoculation of virus

Animal studies were performed in accordance with animal welfare regulations approved by the Institutional Animal Care and Use Committee of Explora Biolabs.

Five to six week old male Hsd:athymic Nude-*Foxn1*^*nu*^ mice (Harlan) were anesthetized by intra-peritoneal injection of a ketamine (100 mg/kg), dexmedetomidine (0.25 mg/kg) and buprenorphine (0.08 mg/kg) cocktail and immobilized in a stereotactic apparatus (Kopf Instruments). Tumor cells (2.5 × 10^5^ cells of GBM CSC line, 010627 expressing FLuc, or U87 FLuc cells in 2 μL cell culture medium) were implanted over a 5-minute period at 2.5 mm medial lateral and 2.5 mm dorsoventral relative to bregma zero coordinates using a micro-drill (Ideal Micro-Drill™) and a Hamilton syringe. The incision was closed with Ethicon 4–0 sutures and tissue adhesive. Anesthesia was reversed with an intra-peritoneal injection of altipamezole (0.1 mg/kg). Virus treatment was started 2–7 weeks after tumor cell implantation by a single intra-cranial injection (2.5 × 10^6^ pfu in 2 μL PBS). Five mice per group were used in the low tumor burden study and nine mice per group were used in the high tumor burden study.

### Luminescence imaging of tumor growth

Nude mice bearing FLuc expressing tumor cells were imaged after being injected intraperitoneally with 120 μL of a 30 mg/mL D-luciferin solution (GoldBio) using an animal imager (Bruker). Quantitation of luciferase signal was carried out using the Molecular Imaging software (Bruker). To determine the trend of tumor growth over time, median tumor signal was used for the large tumor burden setting and median relative tumor signal in the small tumor burden setting. Relative tumor signal is the ratio of tumor signal at a specific time point compared to just before virus inoculation.

### Immunohistochemistry analysis of GBM tumors in mice brains

Dissected brains were fixed in 10% neutral buffered formalin over night, embedded in paraffin, and 5 μm sections were cut. After deparaffinization, rehydration and antigen retrieval was performed with citrate buffer. A custom made rabbit antibody targeting the A27L structural protein of VACV (GenScript) was used for VACV detection in sections as described in Frentzen et al. [34]. Successive sections were stained for BMP-4 using a mouse BMP-4 antibody (Cell Signalling). As a secondary antibody an HRP-conjugated anti-mouse (Vector Laboratories) was used. Detection was performed using the Vectastain Elite ABC reagent and Vector ImmPact DAB Peroxidase substrate (Vector Laboratories) and sections were counterstained with Hematoxylin.

### Statistical analyses

Statistical analyses of mice survival was assessed using the log-rank test. A *P* value of less than 0.05 was considered statistically significant.

## Results

### VACV mediated BMP-4 expression in GBM CSC cultures facilitates differentiation and generates a bystander effect

**Figure 1:**
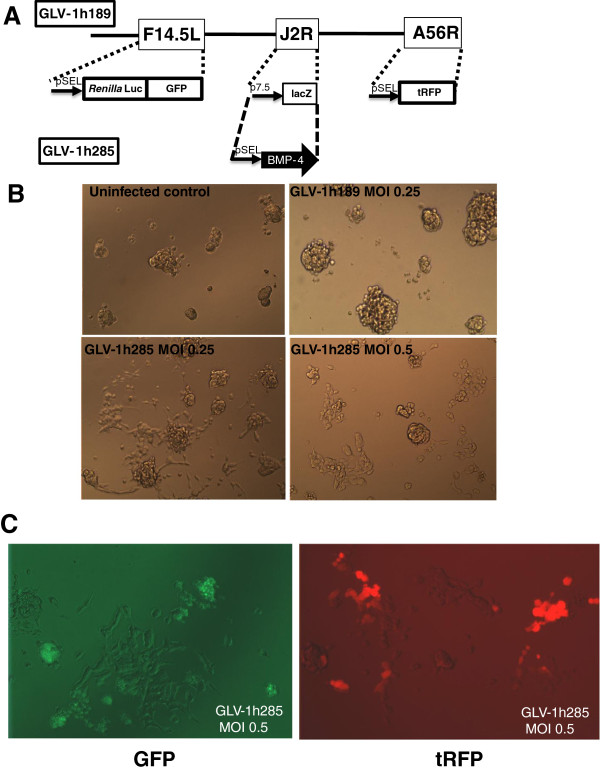
**VACV expressing BMP-4 facilitates differentiation of GBM CSCs. A**) Schematic representation of the two viruses used in the study. GLV-1h189 is the parental virus with insertions of a cDNA encoding a *Renilla* luciferase and GFP fusion in the *F14.5 L* locus, a *lac Z* cDNA in the *J2R* (thymidine kinase) locus and a turboRFP (tRFP) cDNA in the *A56R* locus. To construct GLV-1h285, a cDNA encoding BMP-4 was used to replace the *lacZ* cDNA. The promoters are indicated in front of the boxes representing the cDNAs. **B**) Infection of GBM CSC spheroids with the two viruses. Appearance of differentiated, adherent cells is evident upon GLV-1h285 infection (bottom left) whereas spheroids remain intact upon GLV-1h189 infection (top right) at 9 dpi (Magnification 4X). **C**) BMP-4 generated by GLV-1h285 has a distinct bystander effect. BMP-4 produced from GLV-1h285-infected spheroids (glowing green or red) differentiates the neighboring spheroids resulting in adherent cell clusters at 9 dpi (Magnification 10X).

**Figure 2:**
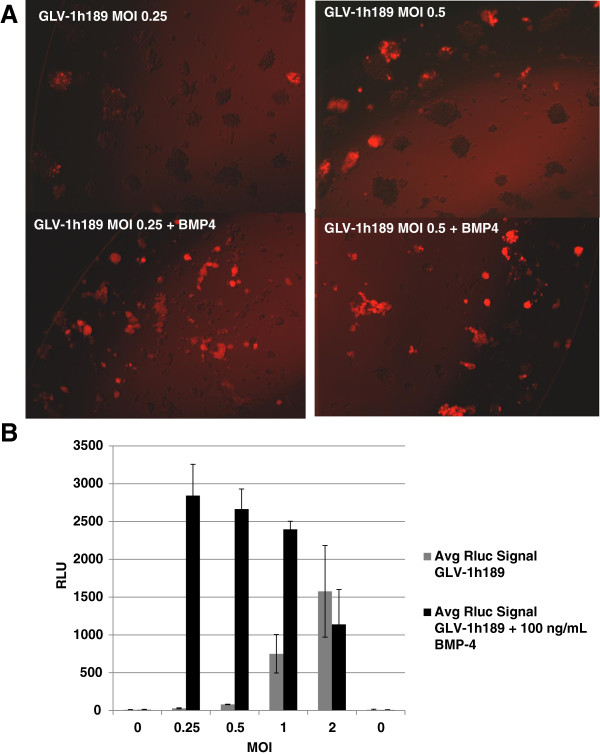
**Purified BMP-4 facilitates enhanced replication of GLV-1h189 in GBM CSCs. A**) GLV-1h189 infection of GBM CSCs in the absence (top panels) or presence (lower panels) of BMP-4 at 9 dpi. In the presence of BMP-4 the majority of the spheroids are broken down into cell clusters that are either infected or have a differentiated appearance. (Magnification 4X). **B**) BMP-4 enhances replication of GLV-1h189 in GBM CSCs as indicated by RLuc expression (relative light units, RLUs) at 9 dpi. GLV-1h189 in the presence of BMP-4 showed heightened replication that ranged from approximately 4–50 fold higher compared to GBM CSCs infected with GLV-1h189 alone at MOIs less than 2. An MOI of 0 is cells only, uninfected background control.

GLV-1h189 is the parental VACV that has three insertions, *Renilla* luciferase-GFP fusion cDNA in the F14.5 L locus, a lacZ cDNA in the TK (J2R) locus, and a turbo RFP (tRFP) cDNA in the HA (A56R) locus (Figure 1A). GLV-1h189 was modified to introduce the cDNA of BMP-4 into the TK locus. Expression of BMP-4 was confirmed by western blotting in both CV-1 cells and GBM CSCs (data not shown). Upon infecting GBM CSC line 010627 (hereafter called GBM CSCs) with GLV-1h189 at an MOI below 1, an average of 30-50% of the culture was found to be infected by VACV, based on GFP or tRFP expression (Figure 2A). Interestingly, a larger proportion of cells were infected at similar MOIs with the virus expressing BMP-4. An intact spheroid architecture was observed for the uninfected cells as well as for cultures infected with GLV-1h189 at all MOIs (Figure 1B, upper panels). However, at an MOI of 0.25, GLV-1h285-infected cultures showed a distinct disruption of the spheroid structures of the GBM CSCs. From a central spheroid-like structure, cells with an adherent morphology, indicative of a differentiated phenotype, emerged (Figure 1B). At a higher MOI of 0.5, a similar differentiated phenotype was evident but with fewer cells in the culture possibly due to loss of cells due to greater oncolytic activity of VACV in differentiated cells (Figure 1B). Interestingly, the adherent cell phenotype was prominent in spheroids that were not actually infected themselves, but close to neighboring infected spheroids, as indicated by GFP and tRFP expression (Figure 1C). Since BMP-4 is a secreted protein this observation is likely due to a bystander effect of protein secretion from spheroids initially infected with GLV-1h285. To further confirm that the morphological microscopic changes were indeed due to differentiation, the expression of glial fibrillary acid protein (GFAP) was monitored. GFAP expression is a well documented marker for GBM stem cell differentiation into astrocytes in response to exposure to BMP [19]. Immunofluorescence observations with a GFAP-specific antibody revealed a heightened level of GFAP expression upon GLV-1h285 infection of GBM CSCs compared to that of GLV-1h189 (data not shown). To confirm that the differentiation phenotype was in fact due to BMP-4 generated from GLV-1h285, an infection of GBM CSCs was carried out using GLV-1h189 in the presence of 100 ng/mL of recombinant BMP-4. As can be seen in Figure 2A GLV-1h189 infection alone resulted in infection of a small proportion of spheroids with no change in the spheroid architecture. However, in the presence of BMP-4, the spheroid like architecture of the GBM CSCs was significantly disrupted, with flat adherent cells emanating from the spheroids. Both the remaining spheroid cells and adherent cells were infected with GLV-1h189, as demonstrated by sharp punctate and diffused expression of tRFP, respectively. Furthermore, visual inspection of the wells infected with GLV-1h189 in the presence of BMP-4 indicated greater tRFP signals compared to wells infected with GLV-1h189 alone at similar MOIs. The RLuc expression from the cDNA introduced in the F14.5 L locus of VACV has been validated as a marker for VACV replication using the VACV maturation inhibitor, ST-246. This inhibitor prevents infectious VACV particle formation. RLuc signal decreased in an ST-246 dose dependent manner upon infection of U-87s cells with GLV-1h189 (data not shown). Therefore quantitative evaluation of RLuc expression, from the wells infected with GLV-1h189 plus BMP-4 indicated a significant increase in viral replication (Figure 2B). This increase in expression was particularly obvious at lower MOIs with an increase of over 2500-fold at an MOI of 0.25.

### BMP-4 VACV infection results in greater cell growth inhibition due to heightened specific replication in GBM CSCs

**Figure 3:**
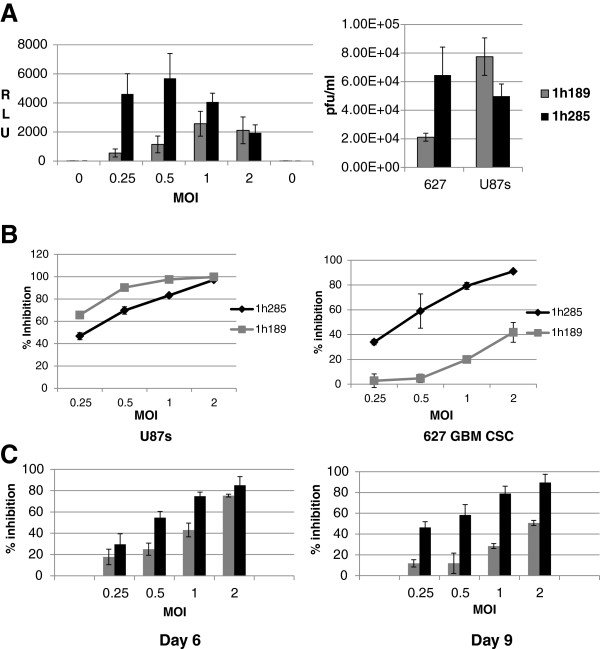
**VACV expressing BMP-4 facilitates growth inhibition by higher levels of replication in GBM CSCs. A**) GLV-1h189 and GLV-1h285 were used to infect the GBM CSCs at different MOIs and replication measured by RLuc expression (RLUs, left panel). Higher levels of replication were observed for GLV-1h285 compared to GLV-1h189, especially at the lower MOIs. An MOI of 0 is cells only, uninfected background control. Plaque assays from infections of the GBM CSCs 010627 (0627) and U87s with both viruses confirmed the RLuc expression data (Figure 3A, right panel). A higher viral titer was obtained for GLV-1h285 compared to GLV-1h189 for the CSC line, but GLV-1h285 did not replicate to a higher titer in case of the U87s cell line. **B**) Growth inhibition assays for GLV-1h189 and GLV-1h285 viruses in the U87s cell line (left) and the GBM CSCs 010627 (0627, right). A higher level of growth inhibition was observed for GLV-1h285 compared to GLV-1h189 in the GBM CSCs. However, similar levels of growth inhibition were observed for U87s for both viruses. **C**) Growth inhibition in the GBM CSCs upon infection with GLV-1h189 and GLV-1h285 at two time points (6 dpi on left and 9 dpi on right). Growth inhibition decreased at 9 dpi in case of GLV-1h189 infection, possibly due to increase in growth of the cells that escaped infection. However, this escape is not observed in case of GLV-1h285 infection.

To determine whether the increase in VACV replication facilitated by purified BMP-4 also occurs when the protein is expressed from the virus itself, GLV-1h285 was used to infect GBM CSCs at various MOIs (Figure 3A, left panel) and RLuc expression determined. As expected, substantially higher RLuc expression was observed for GLV-1h285 compared to GLV-1h189 infections, especially at lower MOIs of 0.25 and 0.5. Furthermore, when the GBM CSCs and a serum-grown glioma cell line adapted to stem cell conditions, U87s, were infected at an MOI of 0.25, a three-fold higher viral titer was obtained from cultures infected with GLV-1h285 compared to those with GLV-1h189 (Figure 3A, right panel). However, for U87s, the production of progeny virus from GLV-1h285 appeared to be slightly reduced compared to GLV-1h189, though close to the range of variability of the assay.

In growth inhibition assays, which examine the viability of cells upon viral infection and expression of BMP-4, the U87s cultures exhibited similar growth inhibition after infection by GLV-1h285 or GLV-1h189 (Figure 3B, left panel). However, in the case of GBM CSCs, GLV-1h285 showed accentuated growth inhibition compared to GLV-1h189 (Figure 3B, right panel) corroborating the higher levels of replication of GLV-1h285 in the GBM stem cell cultures.

To examine the growth inhibition kinetics further in GBM CSCs, an early time point of 6 dpi was included when GBM CSCs were infected with GLV-1h189 and GLV-1h285 at different MOIs (Figure 3C). Differences between the two viruses in growth inhibition were obvious for the early time point with greater inhibition for GLV-1h285, especially at lower MOIs. At the 9 dpi time point, the differences became very pronounced, again especially at lower MOIs.

### Broad spectrum activity and reduced BMP VACV requirement for cytotoxicity across several patient-derived GBM CSC lines

**Figure 4:**
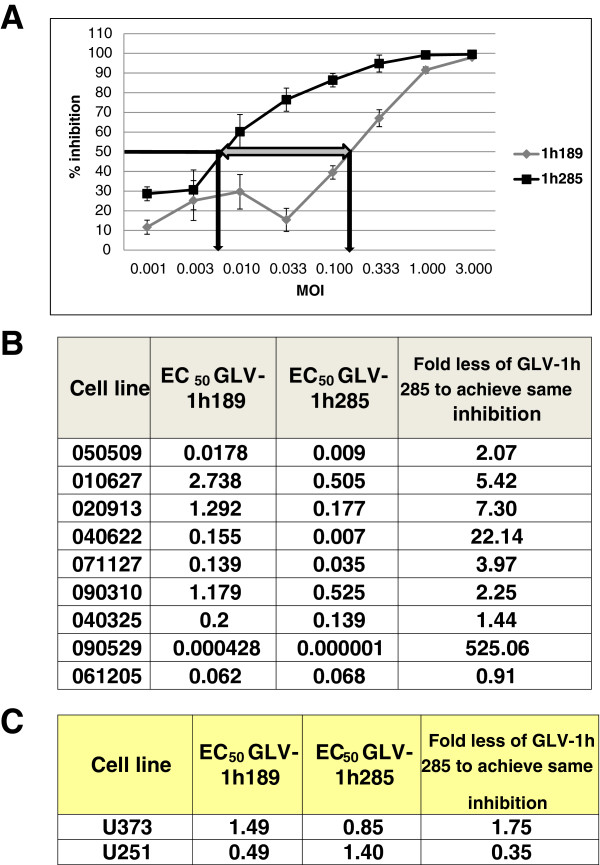
**BMP-4 reduces the amount of VACV needed to achieve growth inhibition in GBM CSCs and has no role in VACV oncolytic activity in glioma serum-grown cell lines. A**) representative growth inhibition curves of GBM CSC line 040622 upon infection with the pair of VACVs, GLV-1h189 and GLV-1h285. The EC_50_ value for each virus is indicated by a black arrow intercepting the X-axis and the difference between the EC_50_ values is shown by the broad double headed arrow. **B**) EC_50_ values for GLV-1h189 and GLV-1h285 upon infection of 9 GBM CSC lines. The fold difference between the EC50 values of GLV-1h189 and GLV-1h285 viruses are in the column on the far right. **C**) EC_50_ values to indicate growth inhibition kinetics by GLV-1h189 and GLV-1h285 in two serum-grown glioma cell lines, U373 and U251 adapted to grow under stem cell conditions.

The activity of GLV-1h285 was tested in eight additional patient-derived GBM CSC lines in growth inhibition assays in parallel with GLV-1h189. As shown in Figure 4A, the EC_50_ values for GLV-1h189 and GLV-1h285, upon infecting the representative GBM CSC line 040622, were quite different with a substantially larger amount (22.14 fold) of GLV-1h189 required for the same degree of growth inhibition as GLV-1h285, suggesting that BMP-4 production might have a general role in facilitating VACV replication in GBM patient samples. Similar tendencies were observed for the majority of the cell lines except 040325 and 061205 (Figure 4B), possibly due to a higher differentiation status of the patient sample from which the cell lines were derived. Further evidence for excluding a role of BMP-4-mediated growth inhibition in differentiated cells in the context of VACV infection came from testing more differentiated cancer cell lines grown in the presence of serum. Two additional serum-grown glioma lines, U373 and U251 were tested with the GLV-1h285 and GLV-1h189 virus pair. Both cell lines showed very similar growth inhibition kinetics for both viruses as indicated by similar EC_50_ values (Figure 4C).

### Intracranial implantation of GBM CSCs forms authentic GBM in brains of immunocompromised mice

In order to develop an orthotopic animal model using the GBM CSCs and to facilitate real time tumor growth measurement, a firefly luciferase (FLuc) cDNA was introduced into the genome of the GBM CSC line, 010627 by lentivirus transduction [20]. This FLuc-expressing variant of the GBM CSC line, 010627 hereafter called GBM FLuc CSCs was stereotactically introduced at specific coordinates in the brains of nude mice. To distinguish tumor growth of the GBM CSC*s* in mice from other conventional serum-grown glioma cells lines, the U87 glioma line was transfected with a plasmid containing the cDNA for FLuc to develop a stable U87 variant capable of expressing firefly luciferase, U87 FLuc. U87 FLuc cells were implanted intracranially similar to the GBM FLuc CSCs.

**Figure 5:**
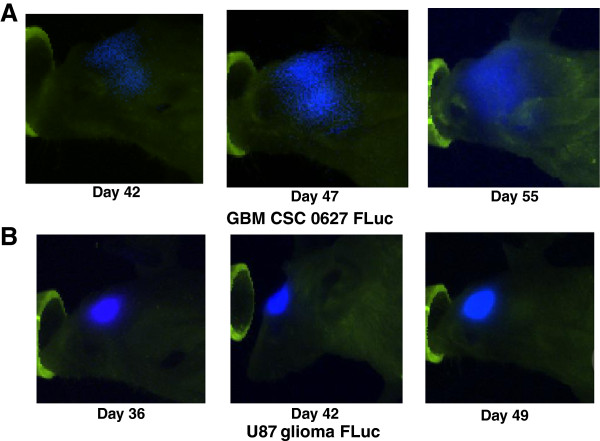
**The GBM CSCs generate an invasive and infiltrative pattern of tumor growth in nude mouse brains reminiscent of glioblastoma in patients. A**) Real time imaging of GBM 010627 FLuc CSC line (GBM CSC 0627 FLuc) after being implanted in the brains of immunocompromised mice. The invasive migratory pattern of cells originating from the site of the GBM CSC implantation (day 42) that can be observed as luminescence (blue) eventually spreads throughout the brain (day 55). **B**) Real time imaging of U87 FLuc cell line after being implanted in the brains of immunocompromised mice. As opposed to the signal pattern from the GBM CSC line, the signal from the U87FLuc cell line (luminescence, blue) remained localized around the site of implantation in the right part of the cerebrum (day 36) and the tumor tended to enlarge only on that side of the brain (day 49). The green color is a false color given to the nose cones for the animals and their fur to generate adequate contrast with the luminescence (blue).

Two to three weeks after implantation an FLuc signal could be detected in the brain for both cell lines upon administration of luciferin. However, as first reported by Galli et al. [4], the pattern of tumor growth was distinctly different for the two cell cultures. The GBM FLuc CSCs start to spread from the site of implantation at right side of the cerebrum to the left side of the cerebrum, via the corpus callosum, at about 42 days post implantation (Figure 5, top). This spread is considered a hallmark feature of GBM in patients [35]. Furthermore, the spread was highly invasive with complete infiltration of the cerebrum occurring within the next two weeks, ultimately appearing like a classical diffused GBM (Figure 5A, day 55, [35]). In contrast, the U87 FLuc cells upon implantation developed a luciferase signal only on the right side of the cerebrum (Figure 5, bottom, 36 days post implantation). The signal grew to some extent over time, but remained localized to the right side of the brain unlike the infiltrative tumor growth observed in GBM patients. By 49 days post implantation the majority of the animals expired mainly due to the build up of intracranial pressure on one side of the cranium.

### VACV mediated BMP-4 expression results in rapid tumor regression and improved survival in immunocompromised mice (low tumor burden)

**Figure 6:**
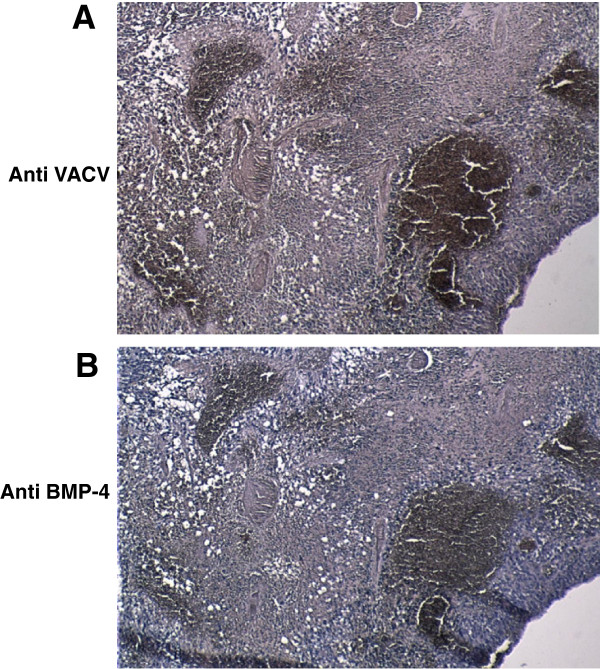
**Colocalization of VACV and BMP-4 staining in brains of GBM xenografts.** Successive paraffin-embedded tumor sections of a GLV-1h285 infected mouse brain implanted with GBM CSCs analyzed by immunohistochemistry, 98 days post injection of the virus. **A**) Detection of VACV structural protein, A27L for identification of areas of tumors infected with VACV. **B**) Detection of BMP-4 expression generated by GLV-1h285. Identification of both proteins in overlapping regions of the tumor is indicative of active BMP-4 production by GLV-1h285 in the GBM CSC xenograft brains.

In order to test the activity of the BMP-4 VACV in the GBM CSC FLuc animal model, GLV-1h285 and GLV-1h189 were injected at the same coordinates as the tumor cells two weeks after implantation in a low tumor burden setting. BMP-4 production could be detected in GBM CSC implants in mice brains upon GLV-1h285 infection by immunohistochemistry analysis using a BMP-4 specific antibody (Figure 6B). The BMP-4 expression was found to coincide with detection of VACV proteins in these mice brains by using an anti VACV structural protein antibody by immunohistochemistry analyses (Figure 6A).

**Figure 7:**
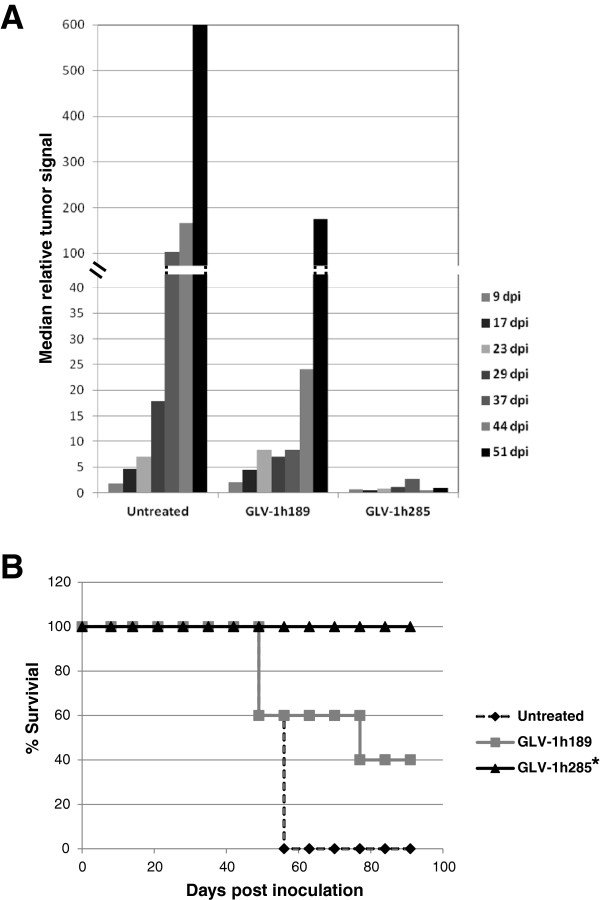
**Rapid and superior tumor regression in GBM CSC implanted mice by BMP-4 VACV translates into improved survival (low tumor burden). A**) Relative tumor signals as measured by intracranial FLuc expression from GBM FLuc CSCs in mice followed by inoculation of GLV-1h189 and GLV-1h285 at two weeks post-implantation. The GLV-1h285-colonized tumors remained at the original size of the point of virus inoculation up to 51 dpi. The GLV-1h189-colonized tumors were controlled until 37 dpi, followed by a rapid increase in tumor size. The untreated controls showed rapid, uncontrolled tumor expansion. **B**) Survival of the implanted and inoculated mice as shown by Kaplan-Meier survival curves. All mice in the untreated group died by day 60. In the GLV-1h189 group, 60% of the mice expired around 80 dpi. However, in the GLV-1h285 group, all mice were alive until 91 dpi. * Indicates P < 0.05 for the GLV-1h285 group when compared with the untreated or the GLV-1h189 groups.

Tumor growth was evaluated in real time by measuring and quantitating FLuc expression on a weekly basis (Figure 7). The untreated tumors grew rapidly and increased in size approximately 670 fold (Figure 7A). In mice inoculated with GLV-1h189 a significant increase in tumor size of up to 175 fold was observed at 51 dpi despite a delay of tumor growth as compared to the untreated control (Figure 7A). In contrast, intracranial administration of GLV-1h285 controlled the tumor size to around or below the initial size, even up to 51 dpi (Figure 7A).

The tumor regression data was found to correspond with survival for the three groups of mice. By 60 dpi, all mice in the untreated control group had either died or had to be euthanized (Figure 7B). Sixty percent of the mice inoculated with GLV-1h189 started to lose weight by 60 dpi and expired soon after (Figure 7B). However, in the GLV-1h285 treated group, all mice were alive until 91 dpi (Figure 7B), indicating a significant survival advantage imparted by viral BMP-4 expression.

### VACV-mediated BMP-4 expression drastically delays tumor progression and improves survival in immunocompromised mice (high tumor burden)

**Figure 8:**
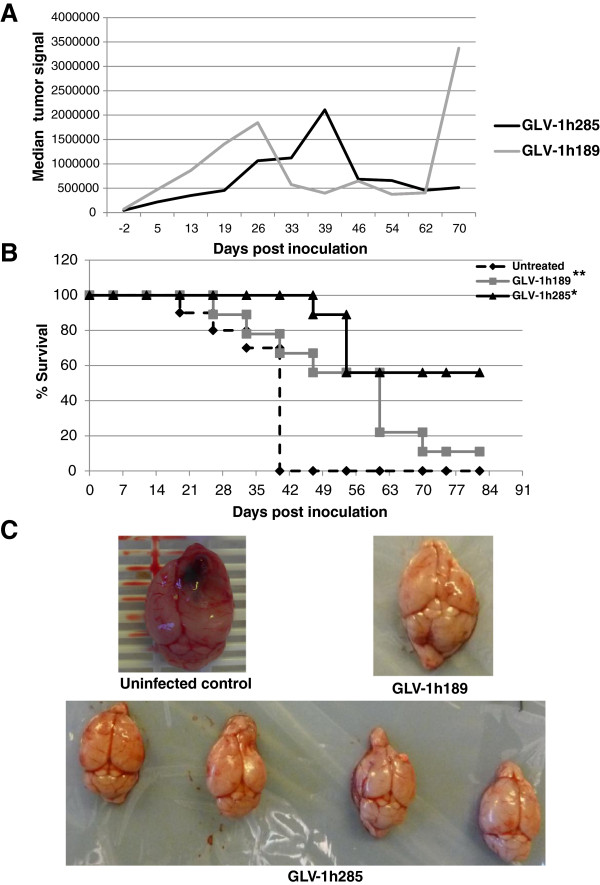
**BMP-4 VACV controls tumor growth and improves survival in mice implanted with GBM CSCs (high tumor burden). A**) Median tumor signals as measured by intracranial FLuc expression from GBM FLuc CSC line implanted in mice followed by inoculation of GLV-1h189 or GLV-1h285 seven weeks post implantation. GLV-1h189-colonized tumors peaked at 26 dpi compared to GLV-1h285 which peaked at 39 dpi. In GLV-1h189-colonized tumors furthermore, at 62 dpi a signal increase due to recurrence of the originally recessed tumor was observed. **B**) Kaplan-Meier survival curve for the experiment described in **A**. All animals in the untreated group died around 42 dpi. A drop in survival was observed for both treated groups of animals albeit later for the GLV-1h285 group following the trend in tumor signal peaks. However, the survival advantage for the GLV-1h285 group was superior with almost 60% of the treated animals surviving compared to only 10% of the animals surviving in the GLV-1h189 group. **C**) Representative whole brains from the mice in the untreated group (top left), GLV-1h189 group (top right) and GLV-1h285 group (bottom). The untreated brain showed an enlarged right half of the cerebrum where severe necrosis and hematoma were observed. The brain from the only surviving mouse in the GLV-1h189 group and representative brains from the GLV-1h285 group show healthy brain tissue. * Indicates P < 0.05 for the GLV-1h285 group compared with untreated or the GLV-1h189 groups. ** Indicates P < 0.05 for the GLV-1h189 group compared with the untreated group.

The efficacy of GLV-1h285 in tumors initiated by GBM FLuc CSCs was also assessed in a higher tumor burden setting. The tumors were allowed to grow for 7 weeks instead of 2 weeks and the viruses were inoculated subsequently. Comparison of the tumor signals after inoculation of GLV-1h189 or GLV-1h285 virus revealed a delay in tumor signal peak for GLV-1h285 compared to GLV-1h189 (Figure 8A). Furthermore, a recurrence of tumor signal was observed only for GLV-1h189 inoculation at 62 dpi onwards, with rapid tumor progression in 80% of the surviving mice. Interestingly, when the survival data was plotted under the tumor signal data (Figure 8B), GLV-1h189 inoculated mice started to expire around 24 dpi with an increase in tumor signal. Another steep decline in survivability was observed at the point where recurrence of tumor signal occurred at 62 dpi. In case of the GLV-1h285 inoculated group, the tumor signal peak also correlated with animal loss. However, it was significantly less than that of the GLV-1h189 inoculated group, with almost 60% of the mice surviving.

Upon euthanasia or termination of the study, the brains of the animals were harvested for examination. Brains from the uninfected group animals showed a high degree of necrosis and hematoma, especially on the right side of the brain where the cells were implanted (Figure 8C top left). Brains from the majority of the GLV-1h285 inoculated mice (Figure 8C bottom) showed substantial improvement in gross morphology compared to the uninfected mice. The few mice that survived after GLV-1h189 inoculation also showed only minor scarring at the site of implantation (Figure 8C top right).

## Discussion

Functional activity of oncolytic viruses is considered to be immune to mechanisms attributed to generate cancer resistance against chemotherapeutic agents and radiation modalities that are considered to reside in CSCs [36]. However, there is a lack of precedence for robust and validated CSC systems to be tested extensively with oncolytic viruses, especially with oncolytic VACVs. The data presented in this study demonstrates the feasibility of designing a VACV that expresses a stem cell differentiation agent, BMP-4 to successfully target infected and non infected undifferentiated GBM CSCs. The resulting effect of a BMP-4 expressing VACV infection causes an enhanced growth inhibition of GBM stem cells *in vitro* and substantial tumor regression in mice compared to the parental, non-BMP-4 carrying VACV.

BMP-4, a member of the TGF-β super family of secreted proteins has been shown to have potential applications in treating GBM and colon cancer [19,30]. However, for making this possible as a treatment modality in patients extensive efforts are required for protein purification. Furthermore, the delivery to the site of action is quite challenging with the protein required to be immobilized on glass spheres or delivered via convection enhanced delivery [19,37,38]. Therefore, expressing BMP payloads from a VACV platform has significant advantages in terms of protein production and delivery in the tumor. In this study we have designed a VACV that successfully expresses BMP-4 and tested this virus in previously validated GBM CSC *in vitro* and animal model systems [4,19].

Quite surprisingly we observed an increase in replication of the BMP-4 VACV in GBM CSC cultures compared to the parental virus and it was found to be specific to the GBM CSC cultures compared to other serum-grown glioma cell cultures (Figures 3 and 4). This is potentially attributed to enhanced second and possibly third round infections facilitated by differentiation by BMP-4 action on the GBM stem cells. Furthermore, the growth inhibition by the BMP-4 virus was substantially greater in GBM CSC cultures compared to the parental virus (Figure 3C). BMP-4 specifically retards GBM cancer stem cell growth [19]. The increase in VACV replication of a CSC culture in the presence of BMP-4 could be due to the ability of the virus to better infect cells that have undergone differentiation. This could result in reduced escape of infection for progeny cells. Hints towards this mechanism of heightened infection and subsequent growth inhibition in the presence of BMP-4 came from the observation that the parental, non-BMP-4 virus infection resulted in reduced growth inhibition at the later time point of day 9 compared to day 6, possibly due to cells that had escaped infection contributing to greater proliferation and reduced growth inhibition (Figure 3C). This phenomenon may simulate the tumor recurrence that is observed in the brains of mice (Figure 8A) and in GBM patients undergoing treatment. However, in the presence of BMP-4 the growth inhibition even increases a little from 6 dpi to 9 dpi for GLV-1h285.

It has been considered that CSCs display potential resistance to infection (replication) by oncolytic viruses engineered for an attenuated phenotype. This was confirmed by our observation that the parental virus infects only 30-50% of the GBM CSC cultures. Elevated interferon levels due to an innate immunity response in CSCs relative to bulk tumor cells is considered to decrease sensitivity to oncolytic virus infection [36]. It would be interesting to determine whether differentiation facilitates lowering of innate immunity and whether that causes an increase in VACV replication in the presence of BMP-4. Additionally the BMP-4 stimulated replication of VACV was more prominent at lower MOIs compared to the parental virus (Figure 3A). This was possibly due to the presence of more viable cells facilitating greater second and third round infections by the virus that expresses BMP-4 and reduced capability of the parental virus to generate substantial infection of the culture at lower MOIs. At higher MOIs for both viruses there was greater parity in terms of replication since fewer cells escape infection. Therefore, differentiation by BMP-4 appears to facilitate infection which can be achieved by using more virus. This higher level of replication for the BMP-4 producing virus, GLV-1h285 results in a lower EC_50_ value indicating the need for lesser amounts of GLV-1h285 to generate the same level of inhibition as the parental virus, GLV-1h189. Furthermore, it appears that the growth inhibition due to GLV-1h189 was by oncolytic activity alone and that of GLV-1h285 due to oncolytic activity by basic VACV infection, growth inhibition by BMP-4 protein and oncolytic activity facilitated by the differentiation carried out by the BMP-4 payload. Evidence for action of the virus generated BMP-4 protein alone comes from observing micrographs where we observed a distinct bystander effect of the secreted protein on GBM CSC spheroids that show a differentiated morphology without being infected (Figure 1C). As was observed in our earlier studies with pure BMP-4 protein and growth retardation of GBM CSC initiated tumors due to differentiation, the differentiated GBM CSCs show significantly reduced proliferation due to decline in number of cell divisions. Interestingly, we observe that the differentiated cells are a better substrate for VACV infection. Whether this happens at the entry step or other stages of the virus life cycle remains to be determined and will be the objective of future studies. However, the three mechanisms of action: basic VACV oncolytic activity of initially infected GBM CSCs, growth inhibition by secreted BMP-4 from these that results in differentiation and facilitation of second and third round infections of the differentiated GBM CSCs resulting in greater oncolytic activity causes significant cellular growth inhibition that translates into tumor growth inhibition in brains of mice implanted with the GBM CSCs.

In case of GBM CSC lines 040325 and 061205, the EC_50_s for GLV-1h285 and GLV-1h189 are very similar, possibly due to a higher level of differentiation of the tumor tissue these lines were derived from. Indeed, in response to exposure to recombinant BMP-4, the 061205 cell line shows reduced growth inhibition compared to other cell lines (Vescovi and Duggal, unpublished). However, this seems to be the exception than the rule among the nine primary cell lines tested, but also indicating the important utility of the basic oncolytic activity of the VACV platform for tumor growth inhibition. Similarly in case of the serum-grown glioma cell lines, U87, U251 and U373, very small differences in growth inhibition were observed between GLV-1h189 and GLV-1h285. As is well documented, growing primary tumor samples under serum conditions selects for a population of cells with a more differentiated phenotype and a genetic makeup different from the original tumor sample [39-41]. Hence, it is not surprising to see lack of superior growth inhibition for the BMP-4 producing virus in differentiated glioma lines since BMP-4 is believed to target undifferentiated, stem cell-like cells. Furthermore, seeing a preference for the BMP-4 virus to replicate and rapidly carry out second and later round infections in the GBM CSC cells is further reassuring as to an undifferentiated, stem cell-like population comprising a significant part of the culture that has a genetic makeup similar to the original tumor [40].

In this study we confirmed in animal xenograft models that the GBM CSCs reproduce the disease much more closely as it occurs in patients [4]. Compared to a representative serum-grown glioma cell line, U87 which remained restricted to one side of the brain, the GBM CSCs migrated to the contralateral cerebral hemisphere possibly via the corpus callosum, a hallmark migratory pattern observed in GBM patients (Figure 5) [4,35]. Furthermore, as is the case with GBM patients the GBM CSC tumors were found to be highly vascular compared to the U87 generated tumors [42,43]. Working with such GBM CSC models could possibly facilitate greater translation of preclinical data in the clinic.

In the GBM CSC animal models we observed a benefit in treating the tumor with the BMP-4 virus without any overt side effects in two different tumor burden settings. Under a low tumor burden setting, the BMP-4 virus caused tumor regression and kept the tumor in check to below the signal when the tumor was first infected up to 51 dpi (Figure 7A). This resulted in significant survival advantage compared to the untreated control group and the parental virus treated group. At a higher tumor burden, the BMP-4 virus delayed tumor growth compared to the parental virus (Figure 8A). Interestingly, the tumor signal of the parental virus treated group showed a rebound after being suppressed from 33 dpi to 62 dpi, a signature event in GBM commonly seen after treatment [44]. However, we did not see a tumor rebound in the BMP-4 virus treated group, supporting the hypothesis that BMP-4 production could disrupt cancer stem cell propagation in GBM.

With CSCs comprising a small population of the tumor there is a concern that the effect of CSC specific inhibitors might not be visible in animal models. Furthermore, this could be reflected in the clinic where the outcome might not register as suitable patient response in terms of tumor growth inhibition as evaluated by classical Response Evaluation Criteria In Solid Tumors (RECIST). Oncolytic viruses on the other hand, with suitable payloads (such as BMPs) to target CSCs could have the ability to register suitable RECIST end points due to their ability to target CSCs, differentiated CSC progeny upon exposure to BMPs and bulk tumor cells. This could consequently increase the chances of observing suitable tumor regression. Additionally, testing oncolytic viruses carrying CSC targeting payloads in diseases such as glioblastoma where the tumor is comprised of a larger proportion of CSCs might have more noticeable effects in a preclinical setting as was observed in the current study. Our study gives the first glimpse of BMP-4 as an efficacious oncolytic virus payload for treating GBM with few side effects. The intracranial delivery of the BMP-4 VACV could possibly be implemented in the clinic in an adjuvant setting similar to what has been done with carmustine wafers after surgical resection [45]. The data presented here also suggests further evaluation of BMPs in combination with other payloads in the context of the VACV platform with a near term goal of testing in the clinic.

## Conclusions

We have used clinically relevant models of GBM using primary CSC-enriched cell preparations to test the activity of a VACV that expresses BMP-4. During this process, we have further confirmed the utility of these primary CSC-enriched systems for drug discovery and introduced real time imaging to monitor effects of the BMP-4 VACV on tumor growth. The BMP-4 VACV was found to have greater levels of replication in these GBM CSC systems compared to the parental virus. This was attributed directly to the expression of BMP-4 which facilitates replication by differentiating CSCs that can serve as a better host for VACV infection. The heightened level of replication and BMP-4 production leads to excellent tumor growth inhibition and survival of mice implanted with GBM CSCs. We believe the data in this article provides a foundation for further evaluation of BMP-4 in the context of VACV replication in combination with other treatments in cancer indications such as GBM in the clinic in the near future.

## Abbreviations

VACV: Vaccinia virus; GBM: Glioblastoma multiforme; BMP: Bone morphogenetic protein; CSCs: Cancer stem cells; TGF: Transforming growth factor; EGF: Epidermal growth factor; FGF: Fibroblast growth factor; NSC: Neural stem cell; GFP: Green fluorescent protein; RFP: Red fluorescent protein; cDNA: Complementary DNA; MOI: Multiplicity of infection; EC50: 50% effective concentration.

## Competing interests

R. Duggal, U. Geissinger, Q. Zhang, J. Aguilar, N.G. Chen and A.A. Szalay are salaried employees of Genelux Corporation and have personal financial interest in Genelux Corporation. No potential competing interest were disclosed by the other authors.

## Authors’ contributions

RD conceived the study, participating in designing the studies, performed all of the CSC culture and most cell culture experiments and wrote the manuscript. UG participated in designing the studies, carrying out majority of animal experiments, histopathology experiments, supported the cell culture experiments and participated in manuscript preparation. QZ prepared the BMP-4 virus construct and participated in manuscript preparation. JA prepared the BMP-4 VACV and parental VACV and participated in manuscript preparation. NGC helped with the VACV production and participated in manuscript preparation. EB helped with the GBM CSC technology and providing stem cell conditions adapted U373 glioma line. AV helped in conceiving the idea for the BMP-4 virus, providing all the GBM CSC technology and participated in manuscript preparation. AAS participated in designing the study, participated in manuscript preparation and approval for submission. All authors read and approved the final manuscript.

## Authors’ information

R. Duggal, U. Geissinger, Q. Zhang, J. Aguilar, N.G. Chen and A.A. Szalay are salaried employees of Genelux Corporation. AV and EB are members of Department of Biotechnology and Biosciences, University of Milan, Italy.
